# Practical Difficulties in Estimating The Prevalence of Primary Infertility in Iran 

**DOI:** 10.22074/ijfs.2019.5583

**Published:** 2019-04-27

**Authors:** Mohammad Mehdi Akhondi, Fahime Ranjbar, Mahdi Shirzad, Zohre Behjati Ardakani, Koorosh Kamali, Kazem Mohammad

**Affiliations:** 1 Department of Embryology, Reproductive Biomedicine Research Center, Royan Institute for Reproductive Biomedicine, ACECR, Tehran, Iran; 2 Nursing Care Research Center, Iran University of Medical Sciences, Tehran, Iran; 3 Reproductive Biotechnology Research Center, Avicenna Research Institute, ACECR, Tehran, Iran; 4 Department of Public Health, School of Public Health, Zanjan University of Medical Sciences, Zanjan, Iran; 5 Department of Epidemiology and Biostatistics, School of Public Health, Tehran University of Medical Sciences, Tehran, Iran

**Keywords:** Epidemiology, Infertility, Iran, Prevalence, Reproduction

## Abstract

**Background:**

According to the World Health Organization (WHO)'s clinical, epidemiological and demographic defi- nitions, infertility is an inability to become pregnant within one, two or five years of exposure to pregnancy, respec- tively. Inconsistent infertility-related definitions and various methodological approaches make it difficult to compare quantitative data in this regard and consequently, have negatively influenced estimating the prevalence of infertility. The present study reviewed the results of a large population-based survey on how the clinical, epidemiological and de- mographic definitions of infertility produce different results in terms of infertility prevalence in Iran and subsequently, compared the findings in order to find the right time of treatment-seeking by couples.

**Materials and Methods:**

This community-based, cross-sectional study was carried out by Avicenna Research Insti- tute in the urban and rural parts of Iran between 2010 and 2011. Using cluster sampling, the reproductive history of 17,187 married women aged 20-40 years, was recorded. Totally, 1011 clusters were randomly selected according to post office codes, proportional to the population of the province. Descriptive and inferential statistical analysis of the data was carried out by SPSS statistical software.

**Results:**

The prevalence of primary infertility based on the WHO’s clinical, epidemiological and demographic definitions were 20.2, 12.8 and 9.2%, respectively. In addition, secondary infertility rate was 4.9%.

**Conclusion:**

Infertility estimates over a two-year exposure period made a 50% decrease in infertility rate; however, increasing exposure period to five years made no significant difference in infertility rate. The findings showed that most of the couples will get pregnant within two years of unprotected sexual intercourse and thus, need no treatment. Due to practical difficulties in estimating the prevalence of primary infertility, the reference limit for time to pregnancy, should be reconsidered and giving more time to younger women to become pregnant, seems reasonable.

## Introduction

According to the World Health Organization (WHO), about 60 to 80 million couples in the world have difficulties in getting pregnant and suffer from infertility as a universally common problem. Obesity, increasing rate of sexually transmitted diseases (STDs) and life style changes increased the prevalence of infertility (1). In the most recent years, the factor of life style was shown to play an important role in decrement of fertility and increment of the use of assisted reproductive techniques (ART) (2). Since infertility may change demographic patterns and lead to economic, social and health complications, different groups of sociologists, epidemiologists and researchers in medical sciences focused on it. In order to understand the magnitude and scope of infertility, it is necessary to consider the infertility definition, socio-demographic context and the study population (3).

Inconsistent definitions of infertility and various methodological approaches make it difficult to compare the quantitative data and have negatively influenced estimating the prevalence of infertility (3, 4). In demography, infertility refers to women who are sexually active and do not use any contraceptive methods but unable to have a live birth. Demographers focus on the end-point of the fertility process because demographic analysis of infertility is often based on secondary data such as the Demographic and Health Surveys (DHS). Although in sociological studies, the most prominent issue is giving birth to a live baby which is a key problem for couples who suffer from infertility, it is clinically important to know whether the woman has difficulties in conceiving or in carrying a pregnancy to term. This different attitude relatively explains the diversity in infertility-related definitions in research and practice (5, 6). Other controversial issue is the time of trying to get pregnant. Based on the clinical definitions, infertility is failure to achieve a clinical pregnancy after 12 months or more of regular unprotected sexual intercourse (7, 8). According to the WHO’s epidemiological and demographic definitions, infertility is an inability to become pregnant within two or five years of exposure to pregnancy, respectively (9). It seems that the exposure time of five years reduces biases and consequently, the fertile population is not classified as infertile (4). Secondary infertility is the inability to bear a child, either due to the inability to become pregnant or the inability to carry a pregnancy to a live birth following either a previous pregnancy or a previous ability to carry a baby to term (9, 10).

In order to avoid over- or under-treatment, the right time of treatment-seeking by couples should be investigated. Not considering this issue may result in unnecessary costs and iatrogenic complication of assisted reproduction such as ovarian hyper-stimulation syndrome (OHSS), multiple pregnancies in the short-term, and shortage of required resources in the long-term. The present study reviewed the results of a large population-based survey on how the definition of infertility affect the infertility prevalence in Iran. The present study also provided the prevalence of primary infertility (in all provinces) and secondary infertility in Iran.

## Materials and Methods

The community-based, cross-sectional study is part of a population-based cross-sectional survey on the reproductive history of Iranian women conducted by the Avicenna Research Institute in 2010 and 2011 in the urban and rural parts of Iran. The study and its written consent form were approved by the Research Ethics Committee of Avicenna Research Institute (No: 29/51/7509). Date of pregnancy, child birth, method of contraception, contraceptive use, stopping and switching contraceptive methods, desire to become pregnant, previous history of abortion or miscarriage, beginning or stopping infertility treatments and divorce were recorded. These data were required for completing the reproductive history of each woman and providing an accurate estimate of the infertility prevalence because measuring continuous exposure to the risk of pregnancy over a period of one year is complicated and detailed information about reproductive history is necessary (11). Before distributing the questionnaire to the participants, it was piloted in three phases. The study sample consisted of Iranian married women aged 20 to 40 years old. We only recruited women aged 20-40 years old to reduce recall bias in taking reproductive history. Overall, the reproductive history of 17,187 women aged 20-40 years was recorded.

We used randomized cluster sampling, in which 1000 clusters were determined based on the proportion of the population in every province. In provinces such as Ilam, Kohgiluyeh and Boyer-Ahmad and South Khorasan, the number of clusters reached 12. Finally, 1011 clusters were determined according to the postal codes and 17 questionnaires were completed in every cluster. Data collection was carried out by 280 trained and qualified interviewers. The interviewers selected households in the field according to postal codes and regional map and recorded the subjects’ demographic characteristics as well as their reproductive history. Written informed consent was obtained from all the participants in this study.

### Statistical analysis

Primary infertility was estimated using a quantitative method based on the reproductive history of participants. Primary infertility was defined as inability to have live birth in women who are sexually active and do not use any contraception after 12 months. To assess the primary infertility rate, the reproductive history of the participants was used as discussed in more detail in previous papers (11, 12). The study data was statistically analyzed using SPSS software (SPSS Inc. Chicago, USA Version 11.5), including descriptive statistics (mean, range, frequency and distribution) and analytic statistics (Chi-square and t test). It should be noted that a P≤0.05 was considered significant.

## Results

The present study included 2216 rural women and 14971 urban women. According to the findings, among 17178 women aged 20 to 40 years old who participated in the study, a total of 456 participants (16.3% of subjects with primary infertility and 3.3% of total number of participants) were infertile until the completion of the interview. Primary infertility rate, based on the definition of infertility in clinical practice, was 20.2% (2783 individuals) and secondary infertility rate was 4.9% (36 subjects). Table 1 shows the prevalence of primary infertility in every province based on the definition of infertility in clinical practice. The prevalence of secondary infertility could not be estimated in each province.

**Fig 1:**
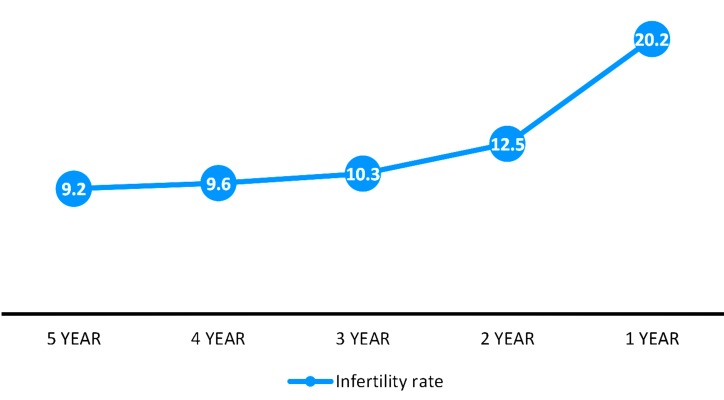
Prevalence of primary infertility according to exposure period to the risk of conception.

Figure 1 depicts the prevalence of infertility based on the period length of exposure to unprotected sexual intercourse that varied from 12 months to 5 years. All seeking-treatment women were considered infertile. We only considered part of this group who got pregnant in the first year after marriage or contraceptive discontinuation, fertile women. With increasing the exposure period to five years, the prevalence of infertility decreased to 9.2% (Fig .1).

**Table 1:** Prevalence of primary infertility in different provinces of Iran based on clinical definition


Name of province	n (%)	Prevalence (%)	2SE

East Azarbayejan	765	21.05	0.21
West Azarbayejan	680	26.24	0.26
Ilam	323	12.04	0.12
Ardebil	1020	33.1	0.33
Isfahan	306	18.05	0.18
Alborz	204	16.81	0.17
Bushehr	221	21.31	0.21
Tehran	3230	18.85	0.19
Cha-harmahal & Bakhtiyari	204	25.17	0.25
South Khorasan	204	36.21	0.3621
Khorasan-e- Razavi	1054	18.96	0.19
North Khorasan	204	18.67	0.19
Khusestan	901	23.89	0.24
Zanjan	238	21.61	0.22
Semnan	204	20.08	0.20
Sistan & Baluchestan	578	11.56	0.12
Fars	884	20.77	0.21
Qazvin	323	17.11	0.17
Qom	340	27.21	0.27
Kordestan	456	14.33	0.14
Kerman	561	18.45	0.18
Kermanshah	425	22.48	0.22
Kohgiluyeh & Boy-erahmad	204	19.77	0.20
Golestan	425	9.52	0.10
Gilan	680	23.81	0.24
Lorestan	408	12.53	0.13
Mazandaran	850	21.23	0.21
Markazi	340	19.05	0.19
Hor-mozgan	340	23.21	0.23
Hamedan	357	23.13	0.23
Yazd	255	21.39	0.21
Total	17187	20.17	0.20


SE; Standard error.

There was a significant difference in age during the first conception attempt between the infertile and fertile groups (P≤0.001). The findings related to the distribution of fertile and infertile couples according to women’s age at time of attempt to get pregnant (no contraception after marriage or contraceptive discontinuation after marriage) are presented in Table 2. It is worth mentioning that 45.0% of the participants were <19 years old on their first attempt to get pregnant. Only 45% of participants with primary infertility (1255) received medical treatments. Among all the study participants (n=1255), the majority (82.6%) were presented first to gynecologists because of infertility problems. It should be noted that referral to specialized centers such as hospitals and infertility clinics was lower than that observed for private gynecology clinic.

**Table 2:** Age distribution of women at the first time of attempt to get pregnant


Womenʼs age (Y)	Fertilen (%)	Infertilen (%)	Totaln (%)

≤14	526 (4.8)	273 (9.8)	799 (5.7)
15-19	4241 (38.5)	1178 (42.3)	5419 (39.3)
20-24	4411 (40.0)	944 (33.9)	5355 (38.8)
25-29	1559 (14.1)	304 (10.9)	1863 (17.2)
30-34	259 (2.3)	72 (2.6)	331 (2.4)
≥35	22 (0.2)	12 (0.4)	34 (0.2)
Total	11018 (100)	2783 (100)	13801 (100)


## Discussion

Generally, infertility and sub-fertility definition is important to manage infertility appropriately (13). Health care providers should be aware of the prevalence of infertility to estimate the likelihood of seeking and undergoing infertility evaluation and treatments. Therefore, there is a need to have a consistent definition for infertility to be used in clinical practice and epidemiological researches (6). In addition to the importance of data collection and analysis to estimate infertility prevalence (11), inconsistency among definitions of infertility across research and clinical practice, can lead to different estimates which makes it difficult to manage infertility.

Also, it should be noted that the infertility prevalence rate in Iran is higher than the global level (12), and infertility has become a national public health problem. Hence, the present study examined the prevalence of infertility in Iran based on different definitions and compared the results subsequently. The prevalence of primary infertility in Iran was 20.2% based on the classic definition used in the clinical practice. Accordingly, one fifth of Iranian couples experienced primary infertility. The results of the present study were similar to other national surveys with regard to the prevalence of primary infertility (based on the classic definition) (14-16). Infertility estimates over a two-year exposure period showed a 50% decrease in infertility rate; however, increasing the period length to five years made no remarkable difference in infertility rate. Infertility estimates over a two-year exposure period in the present study were similar to those reported by Safarinejad (17) in Iran for the same exposure period. Consistently, Gnoth et al. (13) reported that the duration of unwanted non-conception is the main factor in spontaneous fertility.

Mascarenhas et al. (4) suggested considering five-year exposure period for an accurate infertility measurement because longer exposure periods decrease the possibility of the recall bias and are less likely to categorize fertile people as infertile. In another study conducted on infertility, Larsen concluded that the WHO’s epidemiological definition which considers an exposure period of 24 months is more applicable in both research and clinical practice. They divided subjects into infertility and sub-fertility categories and considered those who get pregnant after 12 months and before 24 months as sub-fertile individuals (6). The results of the present study are in line with the results of the Larsen’s study according to which, it seems that considering two-year exposure period produce more accurate results in identifying infertile individuals and is helpful to be used both in clinical practice and epidemiological research. It can be concluded that clinical definition of infertility suggested by the WHO may lead to over-treatment and increase in potentially life-threatening OHSS and multiple pregnancies. Bushnik et al. (18) found that considering questions about the “use of birth control in the previous 12 months”, “the regular sexual activity in the previous 12 months”, and “trying for pregnancy” or “pregnancy intent” in the clinical definition of infertility, results in a lower rate of infertility prevalence.

Unlike the study done by Mascarenhas et al. (4), extending the exposure period to five years made no remarkable difference in infertility rate in the present study. As a result, five-year exposure period cannot be considered a good criterion in the clinical practice or national policy makings. However, delayed fertility in couples trying to conceive for two years is seriously important, and needs thorough examination to understand the causes of infertility.

In addition, women’s age is also an effective issue during the first conception attempt and should be considered in studies estimating the infertility prevalence. There was a significant difference between fertile and infertile couples in terms of women’s age in the first conception attempt. The first conception attempt was defined as not using contraception after marriage or contraceptive discontinuation after marriage. Unlike expectations, the infertile participants were so young (≤19 years old). Over half of the infertile participants were less than 20 years old in their first pregnancy attempt and the number of participants over 35 years old was very few. Although the sample size was too small to draw any relevant conclusions on the effect of higher age on infertility, the effect of womenʼs age as reflected by decreased pregnancy chance among women over 35, cannot be ignored in presenting an appropriate definition for infertility rate. It was suggested that the age range of 19 to 30 years is the appropriate age for Iranian women to conceive and in fact, teenage pregnancy is not suggested due to its risk for mother and baby (12). Gurunath et al. (19) suggested that an appropriate clinical definition should consider both exposure period and the female age. Moreover, Gnoth et al. (13) recommended that a basic infertility evaluation following failure to achieve a pregnancy after 6 cycles, identifies couples with serious infertility problems and may decrease over- or under-treatment despite the age factor. According to this study, couples with good prognosis such as unexplained infertility may be encouraged to wait longer because there is no chance for fertility even through treatment, though others may take advantage of undergoing early ART. Considering the above-mentioned studies, for women of <35 years old, it is suggested to perform a basic infertility evaluation after at least one year and at most two years of unsuccessful conception attempts. For women of <35 with no definitive cause of infertility, it is suggested to continue attempting to conceive and seek medical treatments after two years of trying, whereas for women of >35 years old, it applies after six months of attempting to conceive. Since inability to have a child after two years is a serious issue, it is not reasonable to delay the treatment. Unfortunately, a high percentage of girls in low- and middle-income countries marry before the age of 18 years old. If there is a fertility problem in women aged under 18 years, it will be better to delay fertility treatments.

Based on the present study, current infertility rate (i.e. 3.3%) indicates that about 3 percent of all women of reproductive age have infertility problems and current national facilities, including 70 fertility centers, of the country should support them. Similarly, current infertility rate was reported 3.3, 6.4 and 4.3% in studies conducted by Rostami Dovom et al. (14), and Vahidi et al. (15), respectively. Recent studies reported a secondary infertility rate of about 3 to 8%, which is consistent with the present study (14, 20).

It was reported that just less than half of participants with delayed fertility referred to the specialists for infertility treatments. According to Rostami Dovom et al. (21), about 56% of women with delayed first pregnancy sought to undergo treatment. Boivin et al. (22) reported that 56% of couples with infertility problems were seeking treatment and stated that lack of access or limited access to fertility services is the probable reason for unwillingness to undergo infertility treatment. However, the researchers acknowledged that the demand for infertility treatment is approximately similar in different countries (including developed and developing countries). It is noteworthy that seeking infertility treatment is completely different from giving suitable services. It seems that limited access to appropriate fertility services, high cost of related services and no insurance coverage are among the most important reasons why infertile couples do not refer for the treatment. Out of 70 fertility centers in Iran, over 25 ones are in the capital and other provinces suffer a serious limitation in this regard (23). However, more quantitative and qualitative studies are needed to examine the treatment-seeking behavior among infertile couples in Iran due to cultural and social aspect of infertility, especially considering the stigma associated with infertility in Iran (24). The limitation of present study was including only women of 20-40 years old to prevent recall bias; so, the results might not be extended to all of the infertile women’s population.

## Conclusion

In the present study, we found that most of the participants got pregnant with no infertility treatment over a two-year exposure period and women’s age (≤19 years old) is one of the most important reasons of delayed pregnancy. Due to practical difficulties in estimating the prevalence of primary infertility, it seems that the reference limit for time to pregnancy should be reconsidered in future studies and giving more time to younger women seems reasonable. As the resources are limited, following this policy can greatly reduce costly diagnostic procedures and additional treatments. Since infertility is a serious issue after two years of unsuccessful attempt, it is not reasonable to delay the diagnostic and therapeutic approaches. Caution must be taken in applying these findings to the clinical practice and more studies are required to choose an accurate criterion for both clinical practice and national policy-making.
